# Association between carotid intima media thickness and acute ischemic stroke at an Indonesian tertiary referral hospital

**DOI:** 10.1016/j.jtumed.2022.12.008

**Published:** 2022-12-29

**Authors:** Rivan Danuaji, Suroto Suroto, Bambang Purwanto, Dono Indarto, Faizal Muhammad, Diah K. Mirawati, Vitri Widyaningsih, Soetrisno Soetrisno, Subandi Subandi, Pepi Budianto, Yetty Hambarsari, Baarid L. Hamidi, Hanindia R. Prabaningtyas, Ervina A.J. Hutabarat, Ira Ristinawati, Teddy Tejomukti, Raden A.A. Tedjo

**Affiliations:** a Doctoral Degree Programs of Medical Sciences Department, Faculty of Medicine, Sebelas Maret University, Surakarta, Indonesia; b Neurology Department, Faculty of Medicine, Sebelas Maret University, Surakarta, Indonesia

**Keywords:** السكتة الدماغية, تصلب الشرايين, الحوادث الوعائية الدماغية, سماكة الطبقة الداخلية للشريان السباتي, إندونيسيا, Atherosclerosis, Carotid intima-media thickness, Cerebrovascular accident, Indonesia, Ischemic stroke

## Abstract

**Objectives:**

A high prevalence of tobacco smoking contributes to a high incidence of acute ischemic stroke (AIS) in Indonesia. Large-artery atherosclerosis is known to be a significant cause of AIS. The present study was aimed at evaluating the association between AIS and atherosclerosis on the basis of carotid intima-media thickness (CIMT) measurements in a tertiary care hospital in Indonesia.

**Methods:**

A total of 79 patients with AIS (case study group) and 79 individuals without AIS (control group) were included. Chi-squared tests and odds ratios were used to compare the groups and determine associations. We also considered factors such as age, body mass index (BMI), sex, type-2 diabetes mellitus (T2DM), hypertension, smoking status, dyslipidemia, socioeconomic status, and educational level in the statistical analyses. A p-value <0.05 was considered statistically significant.

**Results:**

Stratification of atherosclerosis into case study and control groups with respect to all study variables indicated a significant relationship (p > 0.05) between atherosclerosis and all variables except low socioeconomic status (p = 0.265) and low educational level (p = 0.180). Regression analysis demonstrated that a BMI ≥25 kg/m^2^, compared with a normal BMI, was associated with a 2.139-fold higher risk of atherosclerosis.

**Conclusions:**

AIS was associated with atherosclerosis, on the basis of CIMT measurements, according to age, BMI, sex, T2DM, hypertension, smoking status, dyslipidemia, socioeconomic status, and education level in the Indonesian population.

## الملخص

**أهداف البحث:**

يساهم الانتشار المرتفع لتدخين التبغ في ارتفاع معدل الإصابة بالسكتة الدماغية الحادة في إندونيسيا. من المعروف أن تصلب الشرايين الكبير هو سبب مهم للسكتة الدماغية الحادة. هدفت الدراسة الحالية إلى تقييم العلاقة بين السكتة الدماغية الحادة وتصلب الشرايين بناءً على قياسات سماكة الطبقة الداخلية للشريان السباتي في مستشفى المستشفيات التخصصية الإندونيسية.

**طريقة البحث:**

إجمالا، تم تضمين 79 مريضا يعانون من السكتة الدماغية الحادة (مجموعة دراسة الحالة) و 79 فردا بدون سكتة إقفارية حادة (مجموعة التحكم). تم استخدام اختبار كاي تربيع ونسب الأرجحية لمقارنة المجموعات وتحديد ارتباطها. أخذنا في الاعتبار أيضا عوامل مثل العمر، ومؤشر كتلة الجسم، والجنس، وداء السكري من النوع 2، وارتفاع ضغط الدم، وعادات التدخين، وعسر شحميات الدم، والحالة الاجتماعية والاقتصادية، والمستوى التعليمي في التحليلات الإحصائية.

**النتائج:**

أظهر التقسيم الطبقي لتصلب الشرايين في دراسة الحالة ومجموعات الضبط فيما يتعلق بجميع متغيرات الدراسة علاقة معنوية بين تصلب الشرايين وجميع المتغيرات باستثناء الحالة الاجتماعية والاقتصادية المنخفضة والمستوى التعليمي المنخفض. أظهر تحليل الانحدار أن مؤشر كتلة الجسم 25 كجم / م 2 كان مرتبطا بخطر الإصابة بتصلب الشرايين بمقدار 2.139 ضعفا من مؤشر كتلة الجسم الطبيعي

**الاستنتاجات:**

ارتبطت السكتة الدماغية الحادة بتصلب الشرايين على أساس قياسات سماكة الطبقة الداخلية للشريان السباتي مع مراعاة العمر، ومؤشر كتلة الجسم، والجنس، وارتفاع ضغط الدم من النوع 2، وعادات التدخين، وخلل شحميات الدم، والحالة الاجتماعية والاقتصادية، ومستوى التعليم لدى السكان الإندونيسيين

## Introduction

Stroke or cerebrovascular accidents are debilitating emergent neurological conditions characterized by acute cerebral hypoperfusion, and are the third most common cause of disability and the second leading cause of death worldwide.^1^ The mortality rate associated with stroke in Asia (except in several Asian countries such as Japan) is higher than that in America, Australia, and Western Europe.^2^^,^^3^ Moreover, no epidemiological data are available to establish the precise incidence of stroke in Indonesia. An Indonesian study has estimated that stroke contributes to 15.4% of all deaths. Its prevalence is 0.022% and 0.0017% in urban and rural Indonesia, respectively. Acute ischemic stroke (AIS) (42.9%) is more common than subarachnoid hemorrhage (1.4%) or intracerebral hemorrhage (18.5%) in Indonesia.^4^

The most common risk factors for stroke in Indonesia include a high prevalence of active tobacco smoking, followed by hypertension and type-2 diabetes mellitus (T2DM).^5^ Active smoking is a significant risk factor for vascular dysfunction, lipid profile alteration, and impaired platelet and endothelial function. The consequent inflammatory processes primarily cause the progression of three atherosclerotic diseases: coronary heart disease, stroke, and peripheral artery disease.^6^

Several longitudinal studies have reported a relationship between smoking and carotid artery atherosclerosis. These studies have measured the carotid intima–media thickness (CIMT) to determine the progression rate of atherosclerosis.^7^, ^8^, ^9^ Furthermore, CIMT or plaque progression is associated with baseline risk factors for atherosclerosis and an elevated risk of stroke.^10^ However, no studies have investigated the association between decreased carotid lumen diameter and AIS in a country with a high prevalence of smoking, such as Indonesia. Given that only urban hospitals in Indonesia have neurosurgical, neuroimaging, and neurological care facilities,^4^ we aimed to determine the association between atherosclerosis (according to CIMT) and AIS in patients at a tertiary referral hospital in Surakarta city, Indonesia.

## Materials and Methods

### Study design and participants

This 2-year (2019–2021) case-control study included patients with AIS at the Neurology Department of Dr. Moewardi Hospital, Surakarta, Indonesia. Patients with AIS who were ≤60 years of age and who exhibited hypodense areas in the brain parenchyma on non-contrast computed tomography scans were included in the study. Furthermore, we included patients who met two or more of the following criteria: (1) hypodense lesions on non-contrast computed tomography, (2) acute onset of weakness in one or more limbs, and (3) decreased consciousness level, with Glasgow coma scale score <15.

The exclusion criteria were patients receiving diuretics or antiplatelet drugs, and those with a medical history of kidney disease, obstructive pulmonary disease, congestive heart failure, chronic liver diseases, lympho-myeloproliferative disorders, or hyperthyroidism or hypothyroidism. Obese patients with a body mass index (BMI) >30 kg/m^2^ and a history of alcohol consumption were also excluded. Patients with AIS were designated as the case study group, whereas those without AIS were designated as the control group.

### Data collection

Neurologists at our center evaluated the patients' brief medical histories and performed physical assessment of each patient, which was confirmed by the patients' companions. They also measured the CIMT at 6 h after the onset of AIS in the case study group. Furthermore, peripheral blood specimens were collected after 12 h of fasting to analyze biochemical profiles on the day of admission.

We used a Philips EPIQ 5 ultrasound machine to measure CIMT. CIMT was measured 2 cm proximal to the carotid bifurcation in the common carotid artery. This double-blind procedure was performed by a qualified neurologist (Indonesian Neurology College of Neurosonology Expert, Certificate No. 015/KNI-PERDOSSI/VII/2015). The normal CIMT for middle-aged individuals is 0.6–0.7 mm. Abnormal CIMT (>1.0 mm) is associated with a high risk of atherosclerosis. Patients with normal CIMT values are placed in the <25th percentile bracket, thereby indicating a low risk of atherosclerosis or carotid plaques. Consequently, common carotid arteries with CIMT <25% are classified as non-atherosclerotic, and those with CIMT ≥25% are classified as atherosclerotic.^11^

We defined hypertensive patients as those with systolic blood pressure ≥140 mm Hg and diastolic blood pressure ≥90 mmHg, and those who took antihypertensive drugs for more than 5 years. T2DM was defined by glycated hemoglobin levels ≥6.5%. Dyslipidemia was diagnosed if the participants met any one of the following criteria: high-density lipoprotein <40 mg/dl, low-density lipoprotein (LDL) ≥100 mg/dl, triglycerides ≥150 mg/dl, or total cholesterol ≥200 mg/dl. Participants who smoked at least two cigarettes every day for 1 year or more were categorized as smokers. Those who had never smoked or had quit smoking at least 1 year prior were defined as non-smokers.

### Statistical analysis

Numerical or quantitative data included age (years), BMI (kg/m^2^), and duration of AIS (h). Categorical or qualitative data included sex, hypertension, dyslipidemia, T2DM, smoking status, educational level, socioeconomic profile, and atherosclerotic status according to CIMT. Qualitative data are presented as categorical data. We categorized the ages of the patients into 30–45 years and 46–60 years. BMI was categorized into <25 kg/m^2^ and ≥25 kg/m^2^. The duration of AIS was categorized into <5 h and ≥5 h.

We used chi-squared tests to assess the association between the case study and control groups with respect to all variables. The associations were considered significant if the p-value was <0.05. Odds ratios (ORs) with 95% confidence intervals (CIs) were calculated to determine the associations between groups. Binary and multinomial logistic regression analyses were performed to construct a mathematical relationship model between all independent variables and atherosclerosis (according to CIMT) after the normal distribution of all variable indices was evaluated through normality testing (K–S test). A p-value <0.05 was considered statistically significant. IBM SPSS Statistics version 22 for Windows (IBM Corp. Armonk, NY, USA) was used to conduct statistical analyses.

## Results

A total of 58 patients who met the study criteria were included and were equally divided into the case study (n = 79) and control (n = 79) groups (Table 1). The study population contained more male than female patients. Moreover, the number of patients with T2DM, hypertension, smoking, dyslipidemia, low socioeconomic status, or atherosclerosis (according to CIMT) was greater than the number of patients without these conditions (Table 2). In the case study and control groups, stratification of atherosclerosis according to socioeconomic status did not indicate a significant association (p = 0.265). However, a significant association (p < 0.05) was observed between atherosclerosis and the remaining variables (Table 3). Binary and multinomial logistic regression analyses (Table 4) indicated that BMI was the only significant independent variable (p = 0.035) that partially influenced the incidence of atherosclerosis. Furthermore, a BMI ≥25 kg/m^2^ was found to be associated with a 2.139-fold higher risk of atherosclerosis than a BMI <25 kg/m^2^.

**Table 1:** Numerical or quantitative data (n = 158).

Variables	n	Minimum	Maximum	Mean ± SD
Age (years)				
Case	79	30	60	46.18 ± 9.85
Control	79	31	60	46.00 ± 8.86
BMI (kg/m^2^)				
Case	79	23.9	29.9	27.29 ± 1.81
Control	79	21.0	27.4	24.26 ± 1.84
AIS duration (h)				
Case	79	2	9	4.62 ± 2.15
Control	79	–	–	–

Case, patients with AIS; control, patients without AIS; BMI, body mass index; AIS, acute ischemic stroke; SD, standard deviation.

**Table 2:** Categorical or qualitative data (n = 158).

Variables	Case (n = 79)		Control (n = 79)	
	Frequency	Percentage	Frequency	Percentage
Age (years)				
30–45	38	48.10%	40	50.63%
46–60	41	51.90%	39	49.37%
Sex				
Male	44	55.69%	46	58.22%
Female	35	44.31%	33	41.78%
T2DM				
Yes	45	56.96%	48	60.75%
No	34	43.04%	31	39.24%
Hypertension				
Yes	51	64.55%	32	40.50%
No	28	35.45%	47	59.50%
Smoking				
Yes	59	74.68%	28	35.44%
No	20	25.32%	51	64.56%
Dyslipidemia				
Yes	46	58.22%	47	59.49%
No	33	41.78%	32	40.51%
Socioeconomic status				
Low socioeconomic status	35	44.30%	42	53.16%
Moderate socioeconomic status	26	32.91%	23	29.11%
High socioeconomic status	18	22.79%	14	17.73%
Education				
Illiterate	21	26.58%	17	21.54%
Primary	12	15.18%	13	16.45%
Secondary	39	49.36%	37	46.83%
Higher	7	8.88%	12	15.18%
Atherosclerosis according to CIMT				
Yes	52	65.82%	20	25.31%
No	27	34.18%	59	74.69%

T2DM, type-2 diabetes mellitus; CIMT, carotid intima–media thickness.

**Table 3:** Association between CIMT and AIS for all variables.

Variable		Atherosclerosis according to CIMT		n	p	OR (95% CI)
		Yes	No			
Age (years)						
30–45	Case	25	13	38	0.001	6.410 (2.353–17.460)
	Control	9	30	39		
46–60	Case	27	14	41	0.001	5.084 (1.971–13.118)
	Control	11	29	40		
BMI (kg/m^2^)						
<25	Case	8	3	11	0.004	7.385 (1.697–32.139)
	Control	13	36	49		
≥25	Case	44	24	68	0.001	6.024 (2.258–16.073)
	Control	7	23	30		
AIS duration (h)						
<5	Case	29	15	44	NA	NA
	Control	–	–	–		
≥5	Case	23	12	35	NA	NA
	Control	–	–	–		
Sex						
Male	Case	26	18	44	0.002	4.093 (1.679–9.978)
	Control	12	34	46		
Female	Case	26	9	35	0.001	9.028 (3.007–27.101)
	Control	8	25	33		
T2DM						
Yes	Case	28	17	45	0.001	6.259 (2.492–15.722)
	Control	10	38	48		
No	Case	24	10	34	0.002	5.040 (1.756–14.463)
	Control	10	21	31		
Hypertension						
Yes	Case	30	20	50	0.001	5.357 (1.949–14.726)
	Control	7	25	32		
No	Case	22	7	29	0.001	8.220 (2.837–23.818)
	Control	13	34	47		
Smoking						
Yes	Case	35	24	59	0.018	3.079 (1.193–7.945)
	Control	9	19	28		
No	Case	17	3	20	0.001	20.606 (5.096–83.315)
	Control	11	40	51		
Dyslipidemia						
Yes	Case	31	15	46	0.001	4.409 (1.848–10.519)
	Control	15	32	47		
No	Case	21	12	33	0.001	9.450 (2.878–31.031)
	Control	5	27	32		
Socioeconomic status						
Low socioeconomic status	Case	25	10	35	0.001	8.000 (2.883–22.203)
	Control	10	32	42		
Moderate socioeconomic status	Case	17	9	26		
	Control	5	18	23	0.002	6.800 (1.894–24.420)
High socioeconomic status	Case	10	8	18	0.265	2.250 (0.536–9.450)
	Control	5	9	14		
Education						
Illiterate	Case	12	9	21	0.180	2.444 (0.654–9.130)
	Control	6	11	17		
	Case	8	4	12	0.002	24.000 (2.251–255.938)
Primary	Control	1	12	13		
Secondary	Case	27	12	39	0.001	6.075 (2.247–16.421)
	Control	10	27	37		
	Case	5	2	7		
Higher	Control	3	9	12	0.048	7.500 (0.921–61.047)
Total	Case	52	27	79	0.001	5.681 (2.855–11.305)
	Control	20	59	79		

Case, patients with AIS; control, patients without AIS; BMI, body mass index; AIS, acute ischemic stroke; T2DM, type-2 diabetes mellitus; CIMT, carotid intima–media thickness; NA, not applicable; OR, odds ratio; CI, confidence intervals.

**Table 4:** Binary and multinomial logistic regression results between all independent variables and atherosclerosis according to CIMT.

	B	Std. error	Wald	df	p	Exp(B)	95% CI of Exp(B)
Age	0.146	0.336	0.190	1	0.663^a^	1.158	0.599–2.236
BMI	0.760	0.360	4.455	1	0.035^a^	2.139	1.056–4.334
Sex	0.588	0.364	2.605	1	0.107^a^	1.800	0.882–3.674
T2DM	0.524	0.342	2.352	1	0.125^a^	1.689	0.864–3.298
Hypertension	0.217	0.346	0.392	1	0.531^a^	1.242	0.630–2.449
Smoking	−0.634	0.364	3.028	1	0.082^a^	0.531	0.260–1.083
Dyslipidemia	−0.365	0.340	1.153	1	0.283^a^	0.694	0.356–1.352
Constant	−0.358	0.507	0.500	1	0.480^a^	0.699	
SE status							
Low	−0.098	0.427	0.053	1	0.818^b^	0.906	0.393–2.093
Moderate	−0.111	0.461	0.058	1	0.810^b^	0.895	0.363–2.208
High	2.594	0.456	41.034	1	0.542^b^	0.591	0.412–2.195
Education							
Illiterate	0.222	0.588	0.153	1	0.696^b^	1.249	0.410–3.803
Primary	−0.263	0.626	0.177	1	0.674^b^	0.769	0.225–2.620
Secondary	0.266	0.519	0.264	1	0.607^b^	1.305	0.472–3.607
Higher	5.201	0.731	20.192	1	0.347^b^	1.104	0.687–3.901
Intercept	−0.238	0.562	0.179	1	0.673^b^		

B, coefficient value; Std. error, standard error; df, degrees of freedom; 95% CI, 95% confidence interval; BMI, body mass index; T2DM, type-2 diabetes mellitus; SE, socioeconomic.

a Binary logistic regression analysis.

b Multinomial logistic regression analysis.

## Discussion

Ischemic stroke occurs when the arterial supply to a part of the brain or the whole brain is blocked or decreased because of atherosclerosis or embolism. In AIS, hypoxia and the death of brain cells may occur within minutes.^1^ Intracranial atherosclerotic disease (ICAD) in major arteries leads to alterations ranging from minor wall thickening to substantial luminal stenosis. Globally, ICAD is the most common cause of AIS. The middle cerebral artery is the most common site, followed by the internal carotid artery.^12^ Risk factors for ICAD can be divided into modifiable and non-modifiable factors. Age, T2DM, and hypertension are independently associated with both symptomatic and asymptomatic ICAD, on the basis of CIMT measurements in Asian and white patients.^13^^,^^14^ Furthermore, the relationship between ICAD and CIMT is relatively mediated by the coexistence of dyslipidemia, obesity, and hypertension.^12^

CIMT can be assessed with carotid duplex ultrasonography (CDU), the only non-invasive screening method allowing for real-time evaluation of intracranial blood flow status. CDU can be performed at the bedside and is relatively cost-effective. Hence, it is reliable for careful examination of dynamic cerebrovascularization.^15^ For AIS settings involving the internal carotid artery and proximal middle cerebral artery, CDU has 90% sensitivity, thus making it a suitable approach for ICAD screening. Moreover, CIMT assessed with CDU has shown 93% specificity, as compared with CIMT assessed with angiography after thrombolysis in AIS.^16^ Given the limited availability and high demand for CDU, CIMT evaluation should be carefully performed by qualified neuroimaging experts in Indonesian tertiary referral hospitals.

In modern medicine, the most common risk factors for atherosclerosis include modifiable risk factors, such as tobacco smoking, obesity, and physical inactivity, and non-modifiable risk factors, such as sex, age, and race.^17^ A previous study has indicated that the annual rates of first ischemic stroke among men 35–44 years of age (incidence of atherosclerosis: 3 per 1000 individuals) increased over 20-fold. Similar annual rates have also been observed in women approximately 10 years later.^18^ Elevated interleukin (IL)-1β levels during the aging process causes arterial stiffness and oxidative stress. Moreover, atherosclerosis is accelerated by inflammasome-containing NOD-like receptors, owing to obesity, insulin resistance, and toxins present in tobacco.^19^

Ischemic stroke is also considered a distinct consequence of dyslipidemia. High LDL and low high-density lipoprotein levels cause cholesterol build-up within plaques. Plaque progression and formation can occur, particularly in patients with hypertension. Subsequently, the exposed cholesterol-rich plaques undergo a thrombogenic process. However, atherosclerosis results from myriad complex interactions and pathways involving macrophages, monocytes, and cellular processes, such as apoptosis, cellular senescence, and protein aggregation.^20^

Hypertension, smoking, and insulin resistance have been shown to be responsible for upregulation of lectin-like oxidized-LDL (ox-LDL) receptor-1 (LOX-1), thus leading to the initiation of atherosclerotic plaque formation.^21^ LOX-1 expression has been found to be stimulated by pro-inflammatory cytokines such as IL-1, IL-10, and tumor necrosis factor-α.^22^ Moreover, LOX-1 causes ox-LDL degradation, which in turn accelerates atherosclerosis.^23^

Several studies have demonstrated an elevated risk of AIS among individuals with low socioeconomic status (an approximately 30% higher incidence). This population is less likely to be aware of the consequences of stroke. However, more evidence is required to corroborate this finding.^24^ A previous study has shown that the risk of AIS, on the basis of mortality rates and disability-adjusted life years, is >3-fold higher among individuals with low than moderate-to-high socioeconomic status.^25^ Lower educational levels tend to be associated with lower socioeconomic status without considering the knowledge regarding AIS.^26^ However, low socioeconomic status was not significantly associated with AIS in our study. We believe that this finding might have been due to the higher rate of illiteracy in the Indonesian population above 40 years of age.^27^

Regression analysis in the present study showed that a BMI ≥25 kg/m^2^ was associated with a 2.139-fold higher risk of atherosclerosis in the Indonesian population. However, the association between obesity and smoking should be considered a major public health issue. A large cross-sectional study consisting of 5254 participants ≥19 years of age has indicated a positive association between smoking and central obesity, as compared with the absence of smoking (OR: 1.30, 95% CI: 1.02–1.67).^28^ Furthermore, the Indonesian Family Life Survey 2014/2015 (12,081 participants) has shown that being overweight to obese and actively smoking tobacco are associated with elevated incidence of chronic multimorbidity (p < 0.05), particularly involving non-communicable diseases such as hypertension, T2DM, stroke, and ischemic heart disease.^29^ Another study has indicated an association between very high BMI and a 2.7-fold higher risk (95% CI: 2.1–3.3) of mortality due to atherosclerosis-associated diseases.^30^

The present study demonstrated that the prevalence of atherosclerosis in patients with AIS was significantly higher than that in individuals without AIS. Atherosclerosis was also associated with age, BMI, sex, T2DM, hypertension, smoking, dyslipidemia, low-to-moderate socioeconomic status, and a primary-to-higher educational level in Indonesian population. Furthermore, a BMI >25 kg/m^2^, compared with a normal BMI, was significantly associated with a 2.7-fold higher risk of atherosclerosis. Hence, a CIMT value ≥25% or >1.0 mm indicates an atherosclerotic artery, which should be considered a risk factor for AIS.

## Source of funding

This research did not receive any specific grant from funding agencies in the public, commercial, or not-for-profit sectors.

## Conflict of interest

The authors have no conflict of interest to declare.

## Ethical approval

This study was approved by the Health Research Ethics Committee of Dr. Moewardi General Hospital (approval number: 1.052/VIII/HREC/2022; date: 15 August 2022; source: https://komisi-etika.rsmoewardi.com/).

## Authors contributions

All authors read and approved the final manuscript. RD, SU, BP, and DI: Conceptualization, data curation, reviewing, research materials, and logistics support. FM: Research investigator, methods, statistical analyses, software, data interpretation, and writing-original initial draft and final manuscript. DKM, VW, SO, SB, PB, and YH: Writing-reviewing and editing. BLH, HRP, and EAJH: Supervision and validation. IR, TT, and RAAT: Data interpretation and reviewing.
